# Establishing PNB-qPCR for quantifying minimal ctDNA concentrations during tumour resection

**DOI:** 10.1038/s41598-017-09137-w

**Published:** 2017-08-21

**Authors:** T. Ehlert, S. Tug, A. Brahmer, V. Neef, F. Heid, C. Werner, B. Jansen-Winkeln, W. Kneist, H. Lang, I. Gockel, P. Simon

**Affiliations:** 1 Department of Sports Medicine, Rehabilitation and Disease Prevention, Faculty of Social Science, Media and Sport, Johannes Gutenberg-University Mainz, Mainz, Germany; 2 Department of Anaesthesiology, University Medical Centre Mainz, Mainz, Germany; 3 Department of General, Visceral and Transplant Surgery, University Medical Centre, Mainz, Germany; 4 Department of Visceral, Transplant, Thoracic and Vascular Surgery, University Medical Centre of Leipzig, Leipzig, Germany

## Abstract

The analysis of blood plasma or serum as a non-invasive alternative to tissue biopsies is a much-pursued goal in cancer research. Various methods and approaches have been presented to determine a patient’s tumour status, chances of survival, and response to therapy from serum or plasma samples. We established PNB-qPCR (Pooled, Nested, WT-Blocking qPCR), a highly specific nested qPCR with various modifications to detect and quantify minute amounts of circulating tumour DNA (ctDNA) from very limited blood plasma samples. PNB-qPCR is a nested qPCR technique combining ARMS primers, blocking primers, LNA probes, and pooling of multiple first round products for sensitive quantification of the seven most frequent point mutations in *KRAS* exon 2. Using this approach, we were able to characterize ctDNA and total cell-free DNA (cfDNA) kinetics by selective amplification of *KRAS* mutated DNA fragments in the blood plasma over the course of tumour resection and the surrounding days. Whereas total cfDNA concentrations increased over the surgical and regenerative process, ctDNA levels showed a different scheme, rising only directly after tumour resection and about three days after the surgery. For the first time, we present insights into the impact of surgery on the release of ctDNA and total cfDNA.

## Introduction

*KRAS* mutation status is crucial for treatment of colorectal cancer patients, as anti-EGFR therapy is ineffective in the presence of *KRAS* mutations^1^. The use of plasma samples as liquid biopsies based on cell-free DNA (cfDNA) circulating in the blood of tumour patients and potentially harbouring tumour derived DNA (ctDNA) has been proposed to replace conventional classification tests on tumour tissue^2^. Furthermore, ctDNA concentrations might have prognostic value in monitoring of tumour recurrence, as increases in ctDNA have been associated with tumour progression and reduced survival^3, 4^.

Different approaches have been presented to detect and quantify ctDNA, such as quantitative real-time PCR (qPCR) using ARMS primers^5^, PNA clamping^6^, LNA primers or probes^7^, blocking primers^8^, HRM analysis^9^, COLD-PCR^10^, and digital PCR methods^4, 11, 12^. Furthermore, genome wide or hot spot sequencing approaches covering huge numbers of possible cancer related mutations have been developed^13^.

Despite enormous technical improvements, relevant obstacles remain. cfDNA concentrations in plasma or serum are typically low; the fraction of the cfDNA originating from the tumour is even lower and may be heterogeneous in mutation status. Therefore, plasma samples at hand might contain no mutated sequences or only in very low copy numbers, even in the presence of a tumour bearing the sought-after mutation^14, 15^. Furthermore, tumour mutation status is dynamic. For example, *KRAS* mutations are known to develop or spread during therapeutic treatment^16^. Additionally, tumour-derived cell-free DNA fragments in the blood plasma tend to be short and are therefore difficult to detect with conventional methods^17^.

This situation imposes the following two challenges on liquid biopsy approaches. First, the measurement needs to be as sensitive and specific as possible. A low sensitivity can lead to false negative results, whereas low specificity leads to false positive results. In the case of *KRAS* mutation analysis, both would result in health threatening decisions and treatment strategies for the patient. Second, the blood volume from which the analysis can be performed needs to be as low as possible to enable repetitive analyses.

To overcome these challenges, we developed PNB-qPCR (Pooled, Nested, WT-Blocking qPCR), a nested qPCR technique that combines two PCR approaches, each selectively amplifying *KRAS* fragments carrying the seven most frequent point mutations of *KRAS* exon 2. The first round PCR employs WT specific blocking primers to enrich mutant fragments only. The second round qPCR uses mutation specific ARMS primers and short LNA probes to amplify short amplicons from multiple pooled first round products. The modifications of the classical qPCR workflow significantly improved limit of quantification (LOQ, see methods) and limit of detection (LOD) several-fold, below the limits of any DNA detection procedure published so far. PNB-qPCR was successfully applied in a study, detecting *KRAS* mutated tumour DNA in colorectal cancer patients during respective tumour surgery.

## Results

### Optimization of PNB-qPCR

The application of the first round PCR with WT blocking primers in a nested setting together with the mutation specific qPCR reduced background noise drastically. In the qPCR, 30,000 WT copies alone generated a false positive signal at a cycle of quantification (Cq) lower than the calculated Cq for one mutated copy. These false positive signals ranged from 0.14 to 24 calculated copies (median 1.76 copies) in the qPCR. These background signals were effectively reduced to a median of 0.04 calculated copies (0.03–0.45 calculated copies) for 30,000 WT copies using the nested qPCR with WT blocker.

The reduced background noise enabled low-copy detection of mutant cfDNA down to one mutant copy per sample for most primer pairs. In the used setting with one mutant copy in a background of 30,000 WT copies, this equates to a detection rate of 0.003% mutant copies. This rate can possibly be even higher but PNB-qPCR was not tested in higher background, as 100 ng per sample is already on the verge of physiological possibility.

The nested qPCR setting also increased the number of possible reruns of the mutation detection with the identical template more than 100-fold. This is because the amount of first round product is sufficient for almost 2,000 second round qPCRs, about 280 for each possible mutation. The numbers of possible reruns of various methods are compared in Table 1.

**Table 1:** Input requirements and specificities of selected detection methods.

	qPCR	nested qPCR	PNB-qPCR	ddPCR (Multiplex)^13^	BEAMing^12^	Capp-Seq.^14^
Required amount of plasma for quantification*	1700–6800 µl	2700–10800 µl	135–540 µl	600–1900 µl	NA	700 µl
Required amount of plasma for detection*	1700–6800 µl	220–860 µl	43 µl	600–1900 µl	110 µl	700 µl
Nr. of possible test reruns**	1	348	279	1–4***	15 (3*5)	1–7
LOQ (copies/quantification)	6.25–50	12.5–50	3.13–12.5	2–7	NA	1
LOQ (% mutant alleles)	0.02%–0.17%	0.04%–0.17%	0.01%–0.04%	0.01%–0.1%	0.01%	0.02%
LOD (copies/detection)	6.25–25	1–4	1	2–7	NA	1
LOD (% mutant alleles)	0.02%–0.08%	0.003%–0.012%	<0.003%	0.01%–0.1%	0.01%	0.02%

The Table summarizes various applications for detection and quantification of mutated DNA fragments, in our case *KRAS*, by providing the calculated minimum amount of plasma for positive test results, the possible number of repetitions of the test, as well as sensitivity of quantification (limit of quantification, LOQ) and detection (limit of detection, LOD). The required amount of plasma describes the necessary input volume into the isolation step for the method to produce reliable results and is determined by the used template volume, DNA concentration, and sensitivity. For the DNA isolation step, we calculated with a variable input volume and an output volume of 50 µl as template for the detection methods. The number of possible reruns results from the required input volume of the methods and possible pre-amplification steps. It describes, how often a test can be repeated to control the results from a given template volume (in this case 50 µl). The results in the three columns to the left refer to the methods presented in this publication. All calculations and percentages of sensitivity are based on numbers presented in the respective literature. *For 1 detection of all 8 variants, given 10 ng cfDNA/ml plasma and 1% ctDNA; formula: LOD/(cfDNA concentration × copies/ng × ctDNA concentration in cfDNA × input fraction of the total template), for PNB-qPCR: 1/10 ng/ml × 290 copies/ng × 0.01%ctDNA × (40/50) = 0.043 ml = 43 µl. ** = Possible number of reruns of the test; Number of final detections of all 8 variants; from 50 µl output from DNA isolation in required concentration. ***Depending on DNA concentration.

However, precise quantification with the nested qPCR was not as sensitive as in the qPCR due to additional variation originating from the first-round PCR. The additional step of pooling five first-round PCR products in the PNB-qPCR decreased the influence of this variance introduced by the first round PCR by averaging the yield across five replicates. The sole increase of template volume in a single first round PCR would also increase the proportional detection sensitivity but simultaneously increase the quantification bias introduced by this step. In contrast, the distribution of the sample into five separate first round PCRs and subsequent pooling strongly reduced the variance induced by the first round PCR and therefore increased the accuracy and sensitivity of quantification at the same time. PNB-qPCR improved the LOQ from medians of 12.5 copies and 25 copies for qPCR and nested qPCR, respectively, to a median of 6.25 copies for PNB-qPCR, ranging from 3.1 to 12.5 copies (Table 1; Supplementary Figs S2–S4).

### Mutation detection

PNB-qPCR was performed in genomic DNA isolated from FFPE samples and all plasma samples taken before surgery. *KRAS* mutations were detected in FFPE samples of three colon cancer patients (G12V (patient 17), G12S (patient 19), G12C (patient 29); Supplementary Fig. S6). These mutations were confirmed in plasma samples of two patients (17 + 19; Supplementary Fig. S7). Additionally, a *KRAS* mutation was detected in the plasma of patient 13 (G12R; Supplementary Fig. S7), a control patient with a history of chronic lymphatic leukaemia previously negative for *KRAS* mutation. ctDNA concentrations were 1.1 ng/ml (0.9% of cfDNA), 0.06 ng/ml (0.32%), and 2.16 ng/ml (4.06%) for patients 13, 17 and 19, respectively. For patient 29 no ctDNA was detected (Supplementary Figs S7+S11).

### cfDNA concentrations

Individual cfDNA levels of all patients increased over the time of the surgical intervention. We averaged cfDNA values within the patient groups for each of the four phases of the surgical process to eliminate inter-individual variation, as described in the methods section. Across all points in time, cfDNA concentrations of mutation positive patients (group M, n = 12) were significantly higher than those of WT tumour patients (group T, n = 60) and WT control patients (group C, n = 40) (P = 0.00001, ANOVA; mean difference M vs T 88.2 ng/ml (95% CI, 49.3–127.1 ng/ml, P = 0.0001) and M vs C 101.0 ng/ml (95% CI, 60.5–141.5 ng/ml, P = 0.000005); both Tukey’s HSD test, Supplementary Fig. S14). The cfDNA concentrations of the two WT groups did not differ significantly.

We subsequently performed separate Tukey’s HSD analyses for group differences at the four surgical phases. These revealed significant differences between groups in phase II, during surgery before resection of the pathologic tissues, (P = 0.036, n = 28, mean difference M vs. T 38.7 ng/ml, 95% CI, −7.6–85.1 ng/ml, P = 0.04; mean difference M vs. C 50.3 ng/ml, 95% CI, 2.1–98.6 ng/ml, P = 0.042, Tukey’s HSD test), and highly significant differences in phase III, during surgery and after surgical resection of the main tumour mass (P = 0.0001, n = 28, mean difference M vs. T 181.4 ng/ml, 95% CI, 94.3–268.5 ng/ml, P = 0.0005; mean difference M vs. C 204.6 ng/ml, 95% CI, 113.9–295.2 ng/ml, P = 0.0001) and phase IV, after surgery (P = 0.007, n = 28, mean difference M vs. T 116.5 ng/ml, 95% CI, 26.8–206.3 ng/ml, P = 0.006, mean difference M vs. C 130.8 ng/ml, 95% CI, 37.4–224.3 ng/ml, P = 0.018, all Tukey’s HSD tests, Supplementary Fig. S14). Groups T and C did not differ significantly at any phase.

### ctDNA

**Figure 1:**
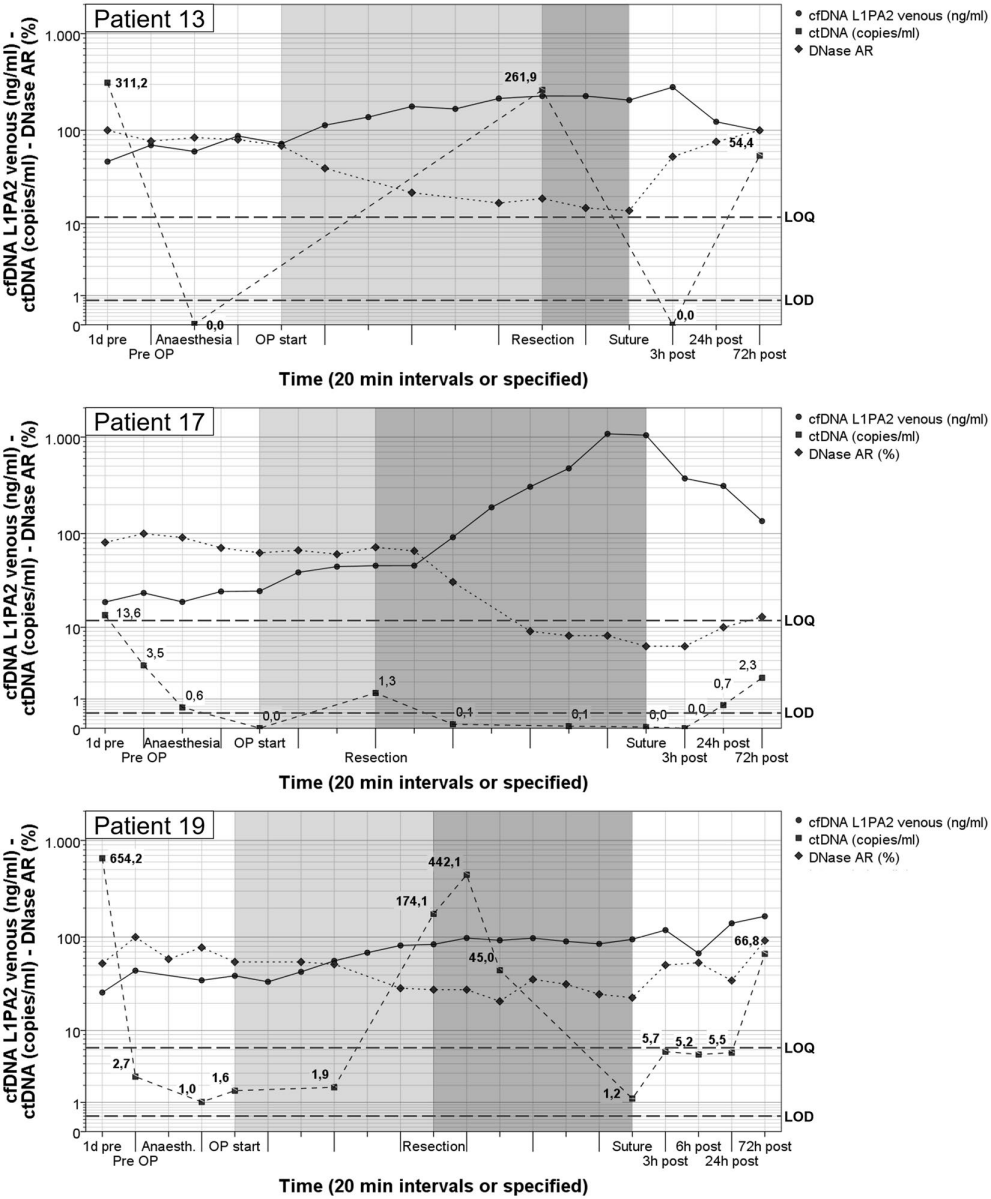
Curve progressions of cfDNA, ctDNA, and DNase 1 activity reduction over the course of surgery. In contrast to the cfDNA values, which steadily increased over the whole course of surgery, ctDNA concentrations decreased sharply during surgery and only increased briefly after resection of pathological tissues and then again gradually in the days following surgery. Changes in DNase 1 AR were inversely correlated to cfDNA development. LOQ and LOD in dashed lines refer to ctDNA values only. Phases of surgery are coloured in grey. Time intervals between samples are 20 minutes if not specified differently. The amount of taken samples was dependent on progress of operation.

For mutation positive patients, selected plasma samples over the period of surgical intervention and the regeneration process were analysed by PNB-qPCR (Supplementary Figs S8–S11). ctDNA concentrations did not correlate with total cfDNA values. However, they showed a similar scheme of progression in all ctDNA positive patients (Fig. 1). ctDNA concentrations were lower directly before surgery than on the previous day. After anaesthesia, ctDNA levels decreased below the LOQ in all cases and below the LOD for patients 13 and 17. Given the low LOD of PNB-qPCR, it can be presumed that no ctDNA was left in the circulation. In all three cases, ctDNA considerably increased directly after the resection of pathological tissue, dropped again within minutes afterwards and re-increased 72 hours after surgery, however, to a lesser extent than directly after resection. The increases directly after tissue resection as well as 72 h post-surgery were observed in all three patients who were positive for *KRAS* mutations in the plasma.

We performed a DNase 1 activity reduction (DNase-AR) ELISA to control for cfDNA decay in blood. Changes in DNase-AR were significantly inversely correlated with cfDNA concentrations across all points in time (two-sided Pearson correlation, ρ = −0.71, P = 10^−8^, for n = 49), as shown in Fig. 1. This confirms a strong connection between the cfDNA concentration and the DNase-AR. However, separate analysis of this dependency over the four surgical phases showed significant correlations for phases II and III only (phase II: ρ = −0.822, P = 0.002, for n = 11; phase III: ρ = −0.761, P = 0.007, for n = 17).

## Discussion

The establishment of plasma DNA analysis as a liquid biopsy is of major interest in cancer research^18^. The suitable way to detect, quantify, and characterize ctDNA depends on the available sample volume and the demands on sensitivity and specificity. qPCR and digital PCR applications are highly sensitive and specific, but limited in information output. NGS applications produce a plethora of information, but are expensive and can be limited in sensitivity^15^. Both generally require large sample volumes.

Here we describe PNB-qPCR, a new cost-efficient application for highly sensitive and specific mutation detection, especially in limited samples. With an LOQ as low as 3 copies for some mutations, PNB-qPCR matches ddPCR, BEAMing, and deep sequencing in quantification and detection sensitivity, requires the lowest input volume and enables more test repetitions from a given sample volume (Table 1). Therefore, PNB-qPCR can produce reliable and reproducible information about the *KRAS* status of every sample, if the sample itself is representative of the *KRAS* status of the patient. This is regardless of the sample size and even works for sample sizes most other methods cannot analyse. PNB-qPCR is also cost-effective and can be performed in almost all labs that possess a qPCR-cycler. Furthermore, PNB-qPCR can be expanded to other targets by a multiplex setting in the first round PCR.

In the future, long-term follow up programs for cancer patients in remission will routinely seek to quantify not only abundance of one particular mutation, but to precisely quantify 10–100 different mutations that have been associated with a specific tumour entity^16^. Moreover, this seems to be particularly necessary, since tumour remissions may be accompanied by other tumorigenic DNA mutations than the ones originally associated with the primary tumour^19^. For this purpose, PNB-qPCR allows the most specific and sensitive analysis for the seven *KRAS* point mutations from minute amounts of plasma volume and will warrant both, robust quantification and saving of sample for analysis of other tumorigenic sequences.

We implemented PNB-qPCR in a clinical trial, analysing ctDNA in serially taken blood samples from cancer patients and matched control patients undergoing colorectal resections. This setting required highly sensitive detection from very limited sample volumes, the hallmark of PNB-qPCR. As the volumes of some plasma samples were lower than 500 µl, few methods could have provided reliable results regarding ctDNA detection and quantification (Table 1).

Total cfDNA concentrations of patients carrying *KRAS* mutations showed higher increases after the resection than cfDNA of WT tumour or control patients, independent of the initial cfDNA concentrations. In contrast, ctDNA concentrations drastically decreased after anaesthesia, and re-increased only temporarily after resection. The initial decreases may have been caused by the administered medication or as a result of fasting. Similar to cfDNA, ctDNA concentrations increased three days after surgery. Knowing these peaks of high ctDNA concentrations enables effective study design for blood withdrawal for subsequent qualitative analysis as a liquid biopsy.

The connection between tumour mutation status and the rapid increases of ctDNA and cfDNA during surgery indicates active mechanisms of DNA release with regard to the observed short time frame. Potential sources of rapid release are extracellular traps, which have been reported to occur shortly after stimulation and depending on tumour status^20^, or microvesicles, which have been reported to contain cancer related DNA fragments^21, 22^. In contrast, the later increases during the regeneration process in the days after surgery were also observed for WT tumour and control patients, to a lower extent however. The time interval and the analogy between the groups suggest apoptotic or necrotic origin for these cfDNA increases^23^. Additionally, we could show a dependency of the rapid cfDNA release during surgery and the DNase-AR that was not observed in the phases before and after surgery. Therefore, cfDNA concentration seems not to be regulated primarily by DNase activity when patients were in a physiologically more steady state.

Here, we successfully established PNB-qPCR, providing a highly effective combination of high sensitivity, high specificity, and improved utilization of limited sample volumes, which is indispensable for ctDNA detection in clinical oncology. Using this technique, we demonstrated for the first time that tumour resection induces rapid release of ctDNA, while increases at later stages can be expected 24–72 h following surgery, revealing ideal points in time for liquid biopsies.

## Methods

### Patients

We recruited 17 patients undergoing tumour resection of the colon (9) or the rectum (8) and a control group of 11 patients with benign diseases matched to the oncologic resections at the University Hospital of Mainz, Germany. The control group was matched to the tumour group for age and sex. In all cancer patients, an R0-status (curative resection) could be achieved and distant metastases had been excluded by preoperative staging. Exclusion criteria were age over 80 as well as severe comorbidities or other conditions that could either bias the study outcome or contradict additional blood withdrawal in the course of surgery for health concerns. One tumour patient (additional to the included 17 tumour patients) had to be excluded from the study during surgery due to diagnosis and resection of two additional tumours in adjacent organs in the surgical process and a resulting re-structuration of the intervention.

The study was approved by the Rhineland-Palatinate’s ethics committee warranting being in line with the Declaration of Helsinki. All patients gave informed consent to participate.

### Sample processing and DNA isolation

Blood samples were collected under standardized conditions one day before surgery, before anaesthesia, under anaesthetic, every 20 minutes during surgery, and 3, 6, 24 and 72 hours following surgery. Blood plasma was separated in two consecutive centrifugation steps within 45 minutes after withdrawal. The centrifugation steps were at 1,600 × *g* for 10 min and 16,000 × *g* for 5 min, respectively.

DNA was isolated from formalin fixed paraffin embedded (FFPE) samples of the solid tumours removed throughout the surgeries using Roti^®^-Histol for deparaffinization and the QIAamp DNA FFPE tissue Kit (Qiagen, Hilden, Germany) following the manufacturer’s instructions. Sample treatment is described in detail in the supplement. Quality and tumour classification of all FFPE samples were confirmed by a pathologist.

### KRAS mutation detection and ctDNA quantification

**Figure 2:**
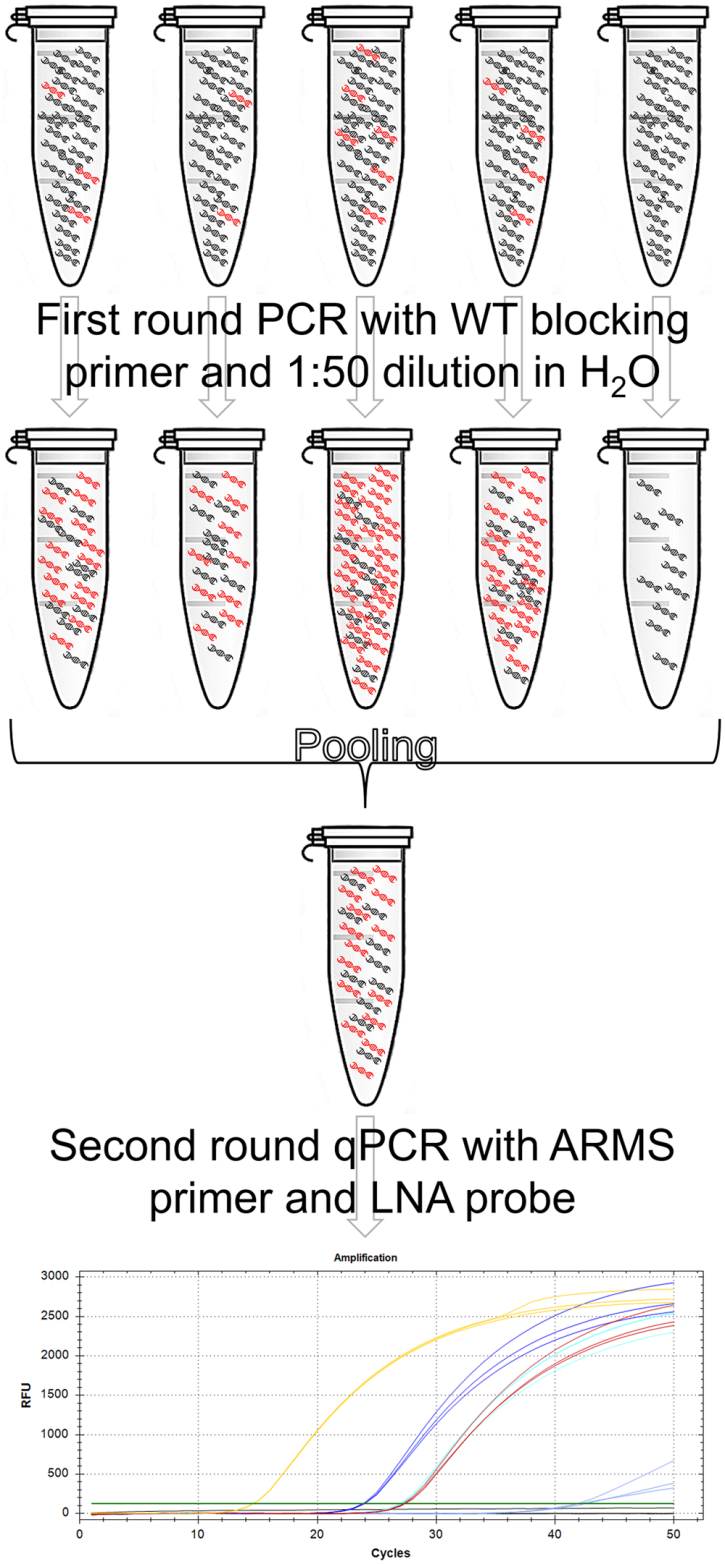
Scheme of the PNB-qPCR. The figure schematically displays the process of the PNB-qPCR with a hypothetical template containing an average of three mutated *KRAS* copies (in red) per PCR in a background of WT DNA (black). The mutated fraction is effectively enriched about 1000-fold. The additional pooling step of five first round products guarantees more accurate quantification in subsequent specific second round amplifications. It reduces variance in target template concentration following first round PCR, especially if low copy numbers of *KRAS* mutations had been present in the starting material.

Mutation detection for *KRAS* was optimized on three levels of PCR complexity, constantly improving the application in specificity, sensitivity, or sample utilization. We determined LOD and LOQ for each a) mutation specific qPCR, b) nested qPCR, and c) PNB-qPCR. The mutation specific qPCR employed ARMS primers specific for the seven most common *KRAS* point mutations and *KRAS* specific LNA probes. LOQ and LOD were defined as the respective lowest DNA concentration that could be quantified or detected with an accuracy of 80% or more in a dilution series of DNA standard fragments, as described in the supplementary material. The nested qPCR incorporates the established second round qPCR as well as a first round PCR using WT outer primers overlapping with the mutation specific inner ARMS primers and a 3′ C6 amine modified WT blocking primer^8^ to enrich mutated fragments, reduce background noise, and enhance the concentration of DNA in the template (Supplementary Fig. S1). PNB-qPCR is different to nested qPCR by pooling quintuplets of diluted first round products containing the same template to eliminate inter-sample variation (Fig. 2).

The first round PCR consisted of final concentrations of 0.002 U/µl Phusion^®^ Hot Start Flex DNA Polymerase, 1x Phusion HF buffer, 0.5 mM MgCl_2_ (NEB, Ipswich, MA), 200 µM dNTPs, 1 µM blocking primer, 400 nM outer primer mix, and 14 µl of template and H_2_O for a total of 25 µl. The first round PCR can be adapted to varying volumes up to 200 µl, permitting up to 112 µl of template. The PCR protocol was performed as follows: 30 seconds at 98 °C, 20 cycles of 10 seconds at 98 °C and 30 seconds at 69 °C, followed by 5 minutes of 72 °C. The PCR product was diluted 1:50 in H_2_O (2 µl product plus 98 µl H_2_O) and served as template for the second round qPCR. Using more than 20 cycles in the first round PCR did not significantly increase the enrichment of mutant fragments, but increased inter sample variation in the second round qPCR and would have necessitated a higher dilution factor, which might have increased variation even further.

For PNB-qPCR, first round PCRs were run in quintuplets. 8 µl of each PCR product were transferred and pooled in a new reaction tube. This pool was subsequently diluted 1:50 in H_2_O for use in the second round qPCR. Figure 2 illustrates the workflow of the PNB-qPCR. Pooling of the first round products was meant to reduce overall variance of the outcome that is partly a consequence of the low template concentrations and partly of running two PCR applications in a row. The second round PCR was a mutation specific qPCR with ARMS primers and LNA probes using final concentrations of 1x SsoAdvanced™ Universal Probes Supermix (Bio-Rad, Munich, Germany), 400 nM forward ARMS and reverse primer, 200 nM LNA probe, and 3.2 µl of template for a total of 8 µl per well. The running conditions were adapted to the specific primer pairs and are described in the supplementary material.

All dilution series for LOD and LOQ determination were created in 1:2 dilution steps ranging from 28.5% to 0.003% mutated DNA, from 12,800 down to 0.8 mutated copies in a background of 30,000 WT copies. Additionally, in each qPCR a no template control containing only 30,000 WT copies was run to control for possible contaminations. All qPCR measurements were performed in triplicates and qPCR measurements were excluded and repeated if the standard deviation was 0.4 cycles or higher. However, no positive results for PNB-qPCR had a higher standard deviation than 0.36 cycles and no concentrations above the LOQ had higher standard deviations than 0.25 cycles.

### cfDNA quantification

Total cfDNA was quantified by three different qPCR approaches. The first one was targeting the repetitive element *L1PA2* in unpurified plasma^24^. The other two were targeting total *KRAS* and *LTR5* ^25^, another repetitive element, respectively, in isolated cfDNA. The results were in very high agreement (Supplementary Fig. S12).

### DNase-1 activity reduction

A DNase 1 activity reduction ELISA assay (ORGENTEC, Mainz, Germany) was performed to investigate whether the changes in cfDNA concentration might be reflected in DNase 1 activity. The assay was performed following the manufacturer’s instructions with plasma of all samples of ctDNA positive patients that had been analysed by PNB-qPCR.

### Statistics

Following mutation detection in the blood plasma, the patients were divided into three groups according to their tumour and plasma mutation status. These groups were patients with *KRAS* mutations in their plasma (group M), as well as tumour patients (group T) and control patients (group C) both without *KRAS* mutations in the plasma. Also, we classified the samples into four main periods of the surgical process, namely period I) pre-surgery, period II) surgery, pre-resection, period III) surgery, post-resection, and period IV) post-surgery.

Statistical calculations for total cfDNA were carried out with the *L1PA2* results using JMP version 8 (SAS Institute) for the Tukey’s HSD tests and SPSS version 22 (IBM) for Pearson correlations. In all tests, P values below 0.05 were regarded as statistically significant.

We calculated mean cfDNA concentrations for each patient for each of the four phases of the surgical process because the number of samples varied between the patients, especially for phases II and III depending on the length of the surgical procedure. Thus, individual variations due to sample numbers and also due to diverging concentrations of single samples were reduced. As the averaged cfDNA values were not normally distributed, they were logarithmized to reach normal distribution and equal variance between groups prior to analysis.

We applied multifactorial analysis of variance for the cfDNA concentrations as main output variable with patient group (n = 3) and surgical phase (n = 4) as independent influencing factors randomizing for “patient” to achieve a paired comparison of data across the four surgical phases. Post hoc tests between groups were done using the all-pair Tukey’s HSD. Correlations between the methods to quantify cfDNA and between cfDNA concentrations and DNase-1 AR were performed using two-sided Pearson correlations following log-normalization.

### Data availability

The datasets generated during and/or analysed during the current study are available from the corresponding author on request. As there are many different datasets included into this publication, we did not generate a publicly available dataset.

## Electronic supplementary material

Supplementary Information
