# Supplementary material for: Establishing PNB-qPCR for quantifying minimal ctDNA concentrations during tumour resection
Source: Sci Rep. 2017 Aug 21;7:8876. doi: 10.1038/s41598-017-09137-w (PMC5566323; doi:10.1038/s41598-017-09137-w)
Supplement: Supplementary file 1 — Supplementary Information [file 41598_2017_9137_MOESM1_ESM.pdf]

Establishing PNB-qPCR for quantifying minimal ctDNA concentrations during tumour resection

–

Supplementary material

T. Ehlert[1], S. Tug[1], A. Brahmer[1], V. Neef[2], F. Heid[2], C. Werner[2], B. Jansen-Winkeln[3,4], W. Kneist[3], H. Lang[3], I. Gockel[3,4], and P. Simon[1]*

[1]Department of Sports Medicine, Rehabilitation and Disease Prevention; Faculty of Social Science, Media and Sport; Johannes Gutenberg-University Mainz; Mainz; Germany

[2]Department of Anaesthesiology, University Medical Centre Mainz; Mainz; Germany

[3]Department of General, Visceral and Transplant Surgery, University Medical Centre, Mainz, Germany

[4]Department of Visceral, Transplant, Thoracic and Vascular Surgery; University Medical Centre of Leipzig; Leipzig; Germany

Supplementary Figures

```
KRAS G12D mutation
5‘ ggcctgctgaaaatgactgaatataaacttgtggtagttggagctgAtggcgtaggcaagagtgccttgacgatacagctaattcagaatcattttgtggacgaatatga 3‘
5‘ GGCCTGCTGAAAATGACTGAATATAAACTTGTGG 3‘                                                         Outer forward primer
Blocking primer       5’ ATAAACTTGTGGTAGTTGGAGCTGGTGGCG[3’-6Amin] 3’
G12D ARMS primer        5‘ AAACTTGTGGTAGTTGGAGCAGA 3‘
WT forward probe                             5‘ aCcgcaTCcgTTctc 3‘
WT reverse probe                               3’ GCGTAGGCAAGAGTG 5’
WT reverse primer                                             3‘ GGAACTGCTATGTCGATTAAGTCT 5‘
Outer reverse primer                                                     3‘ GTCGATTAAGTCTTAGTAAAACACCTGCTTATACT 5‘
3‘ ccggacgacttttactgacttatatttgaacaccatcaacctcgaccaccgcatccgttctcacggaactgctatgtcgattaagtcttagtaaaacacctgcttatact 5‘

Sense strand: Hotspot codon, MUTATION; ARMS primer: Mismatch base, mutated base; LNA probes: LNA bases
```

1st rd. (Outer forward primer, Blocking primer); 2nd rd. (G12D ARMS primer, WT forward probe, WT reverse probe, WT reverse primer); 1st rd. (Outer reverse primer)

Supplementary Figure S1. Priming scheme for PNB-qPCR

Primer and probe constellations including the first round WT blocking primer are displayed regarding their position on the *KRAS* sense and antisense strands.

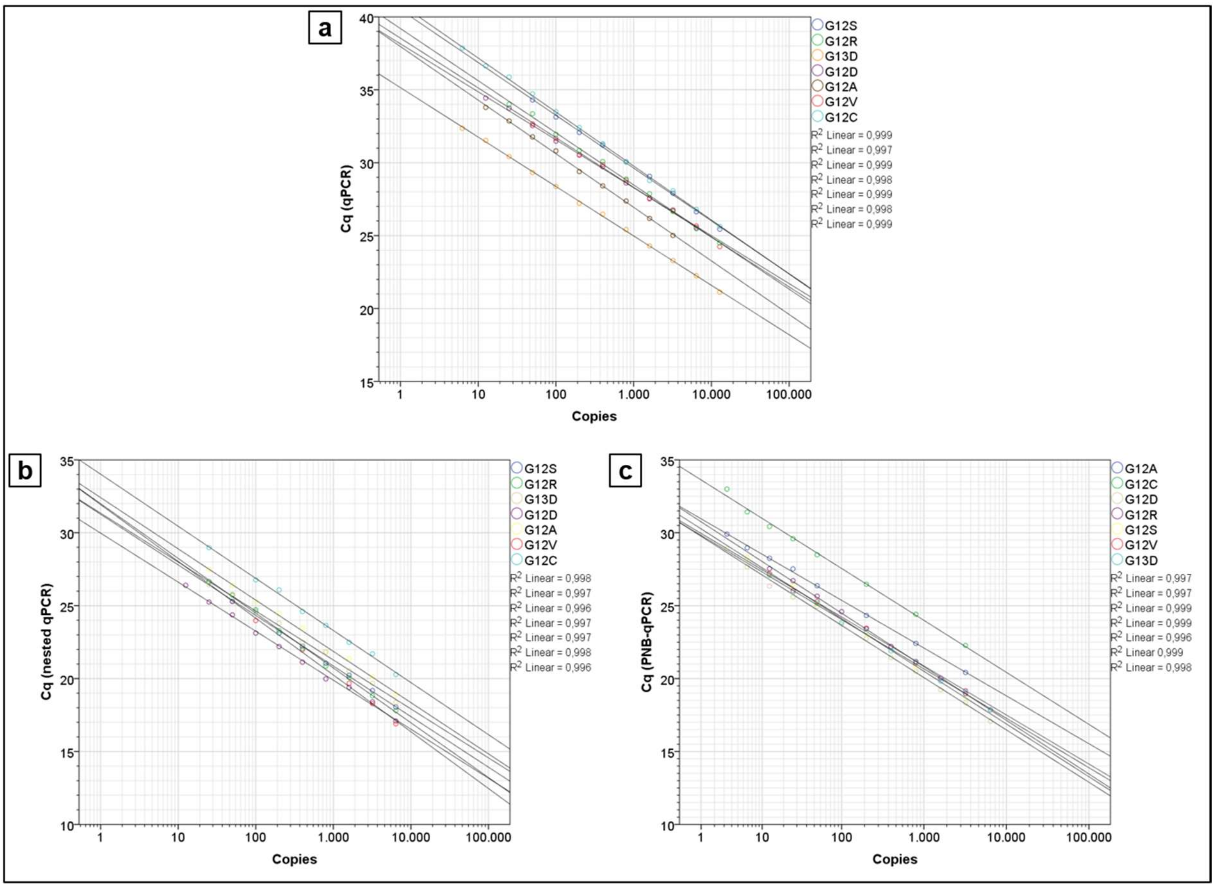

Supplementary Figure S2. LOQ curves grouped by PCR method

All LOQ-Measurements for all seven point mutations in codons 12 and 13 in *KRAS* exon 2, sorted by the three used qPCR applications qPCR (a), nested qPCR (b), and PNB-qPCR (c).

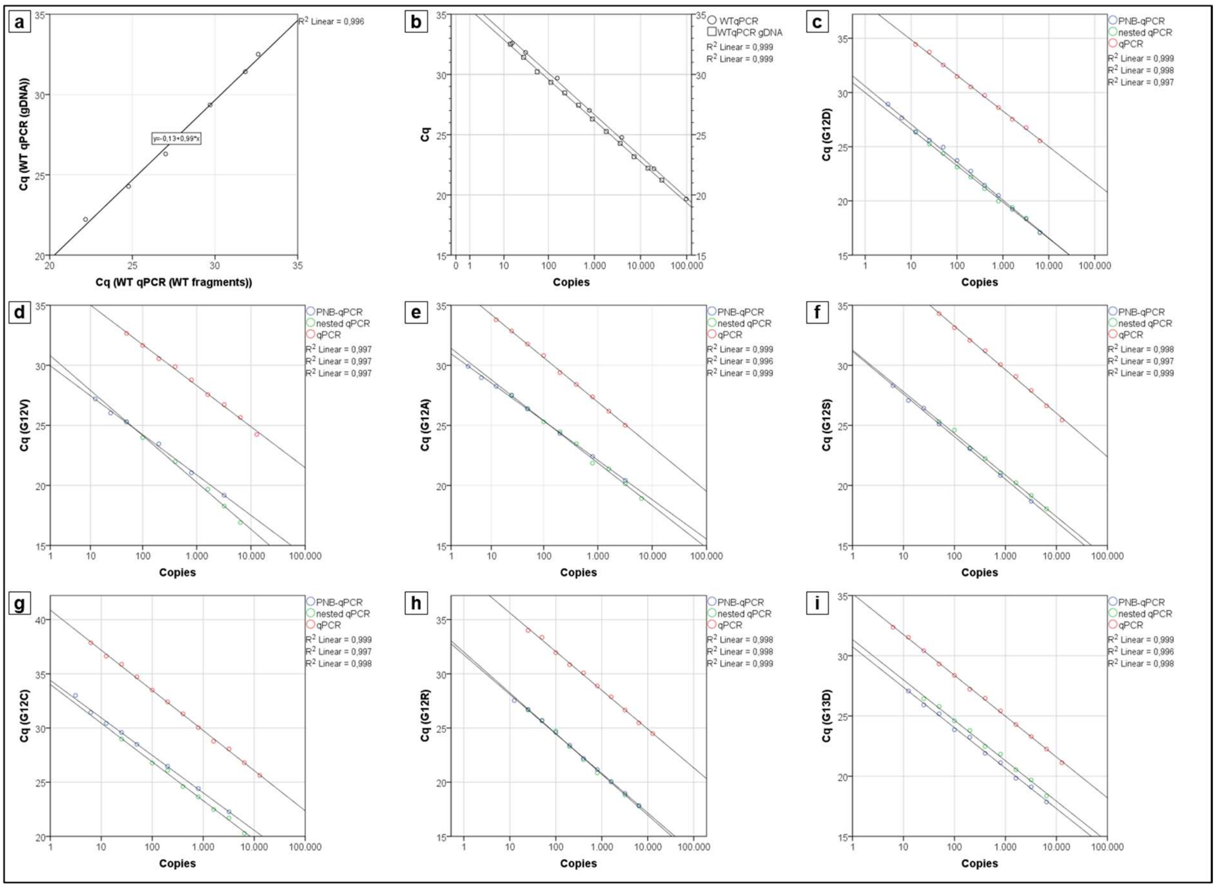

Supplementary Figure S3. LOQ curves grouped by point mutation (c-i) or WT (a, b)

All LOQ-Measurements combined for the three applied methods (qPCR, nested qPCR, PNB-qPCR), ordered by the seven point mutations or WT. Results of WT qPCR dilution series with gDNA or artificial WT fragments highly correlate and are almost identical (a, b). Pre-amplification in a first round PCR with a WT blocker strongly lowers the Cq values of the dilution series (c-i). Pooling five first round products lowered the LOQs fourfold on average.

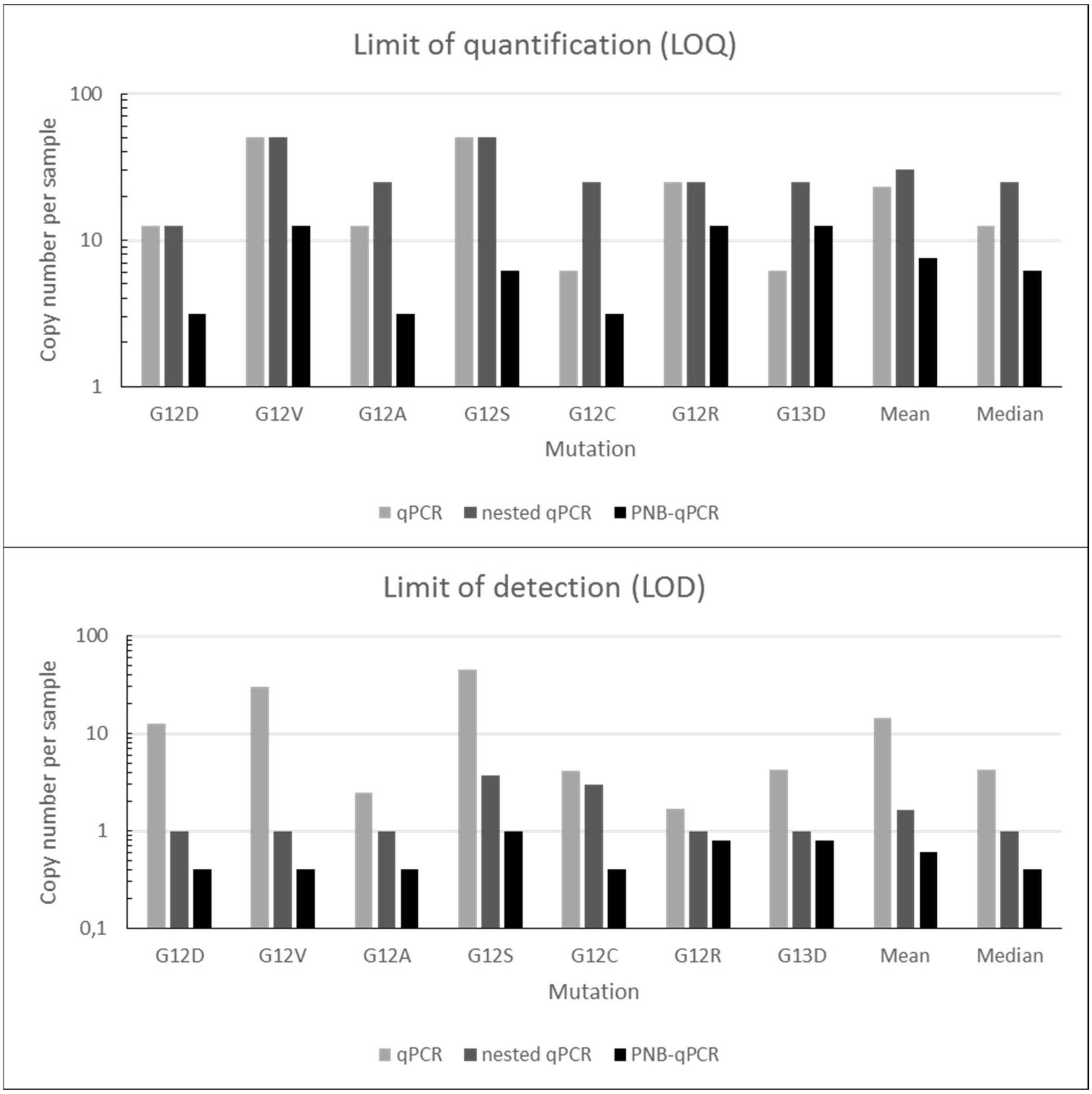

Supplementary Figure S4. Limits of quantification and detection of the seven *KRAS* mutations

The LOD was improved by both the nested setting and by pooling the first-round products in every case down to a median below one copy per first round PCR. The LOQ was not improved by the nested setting but impaired in three cases (G12A, G12C, G13D). PNB-qPCR improved the LOQ in all cases but one compared with the qPCR to a median of 6.25 copies.

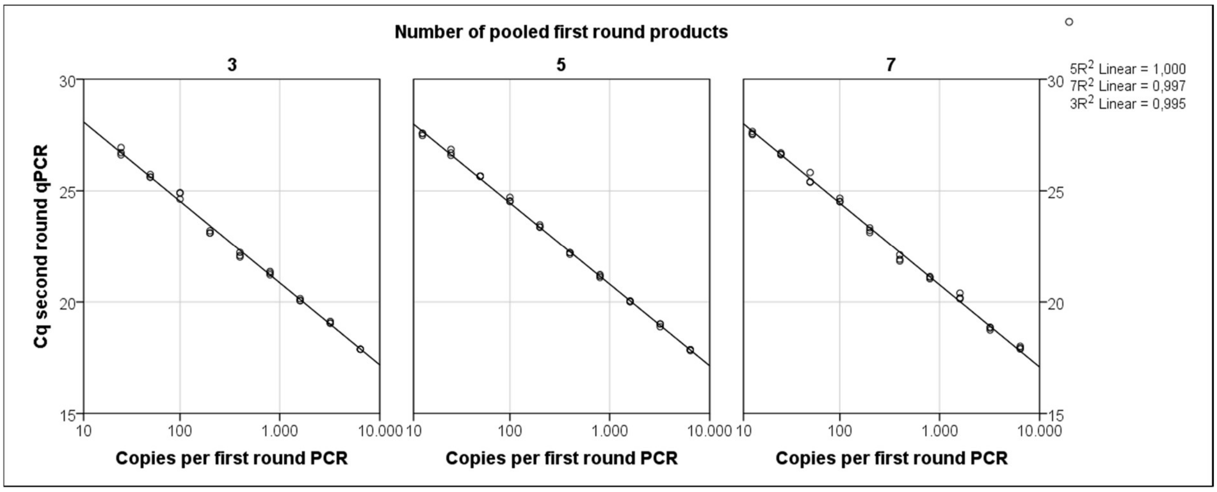

Supplementary Figure S5. Comparison of different numbers of pooled first round products

Pools of only three first round PCRs (LOQ of 25 copies in this case) were inferior to pools of five or seven first round PCR replicates (LOQ of 12.5 copies each). There was no detectable difference between pools of five or seven first round replicates.

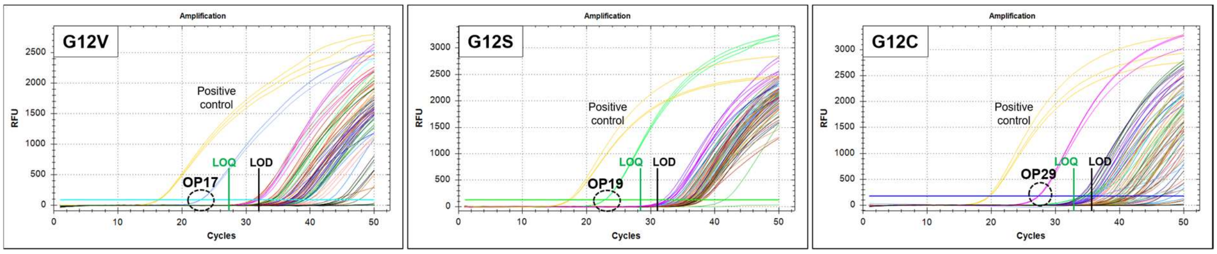

Supplementary Figure S6. The three positive results of PNB-qPCR in the FFPE samples

Positive results were obtained for G12V (patient #17), G12S (patient #19), and G12C (patient #29). All three are colon cancer patients.

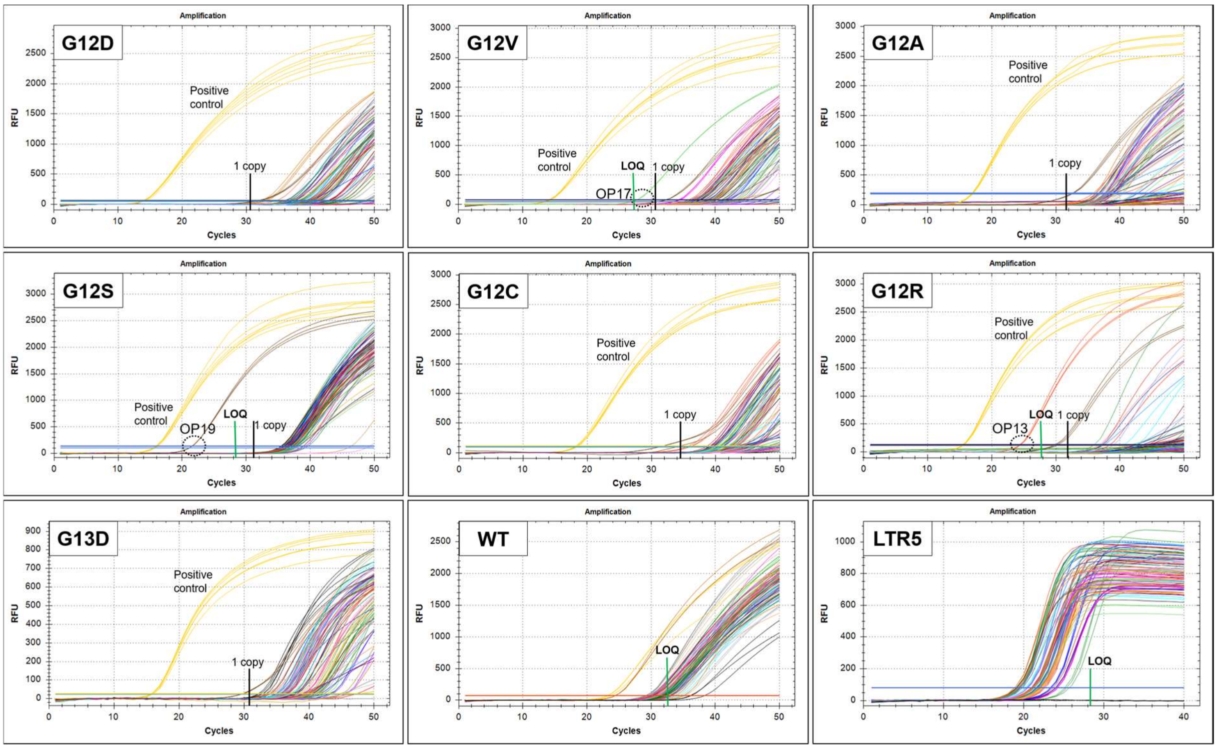

Supplementary Figure S7. Plasma results of PNB-qPCR

Mutation positive results were confirmed for G12V (patient #17), G12S (patient #19). Additionally, a G12C mutation was detected in the plasma of patient #13, a control patient with CLL.

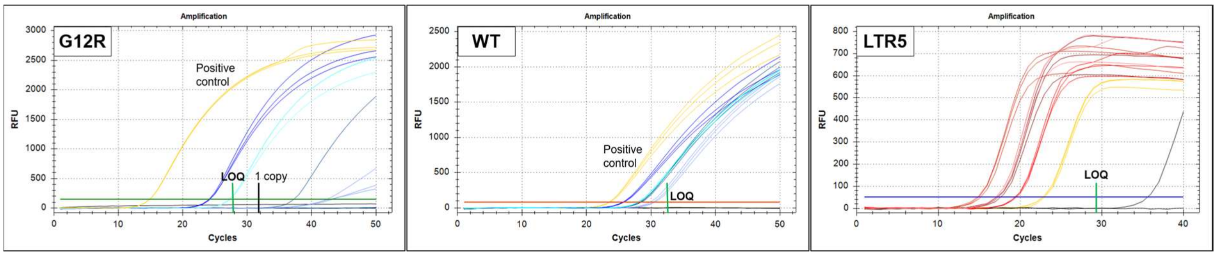

Supplementary Figure S8. Monitoring of ctDNA and cfDNA over surgery – patient #13

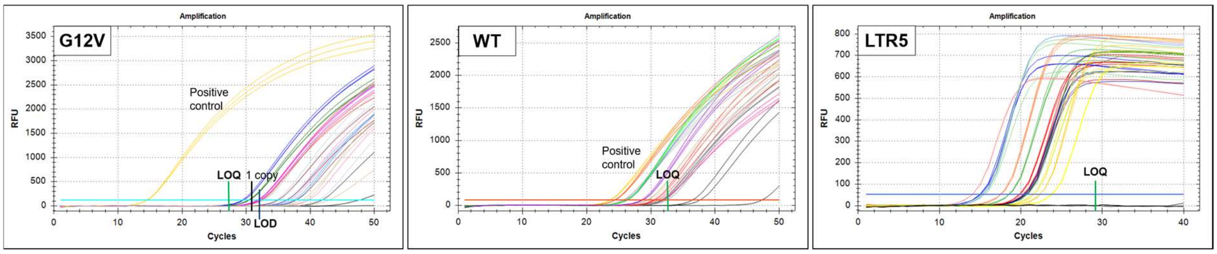

Supplementary Figure S9. Monitoring of ctDNA and cfDNA over surgery – patient #17

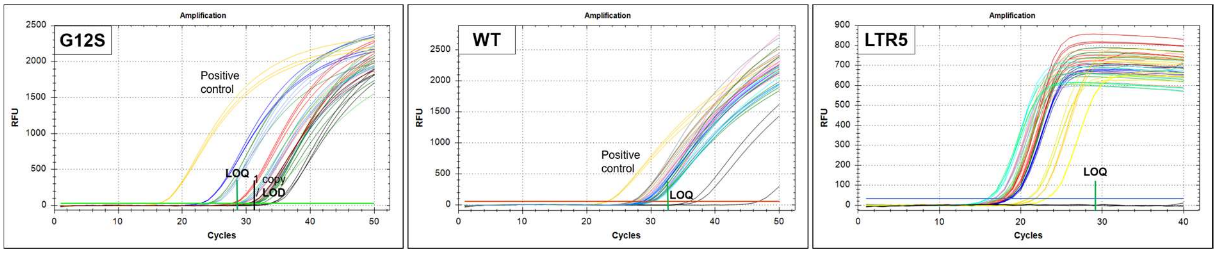

Supplementary Figure S10. Monitoring of ctDNA and cfDNA over surgery – patient #19

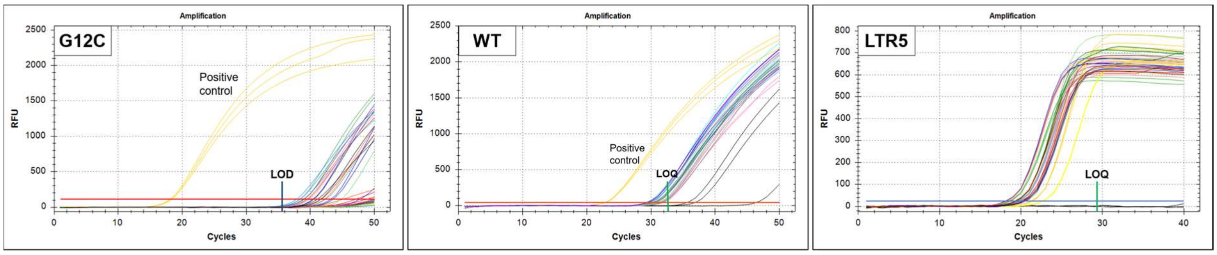

Supplementary Figure S11. Monitoring of ctDNA and cfDNA over surgery – patient #29

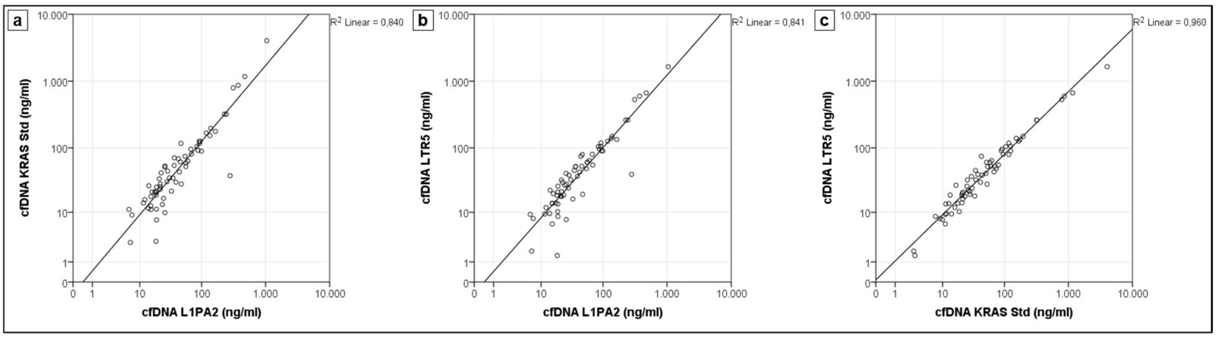

Supplementary Figure S12. Correlations of the WT cfDNA methods

The three used qPCR approaches for total cfDNA are in high agreement. *LTR5* and *KRAS* qPCRs using isolated cfDNA had the highest correlation (2-sided Pearson correlation, $\rho = 0.98$, $P < 10^{-44}$ for 65 comparisons), correlations of *L1PA2* results directly from plasma with *LTR5* and *KRAS* results were interchangeable (2-sided Pearson correlations, $\rho = 0.89$ and 0.87, $P < 10^{-22}$ and $P < 10^{-20}$ for 65 comparisons of *L1PA2* with *LTR5* and *KRAS*, respectively).

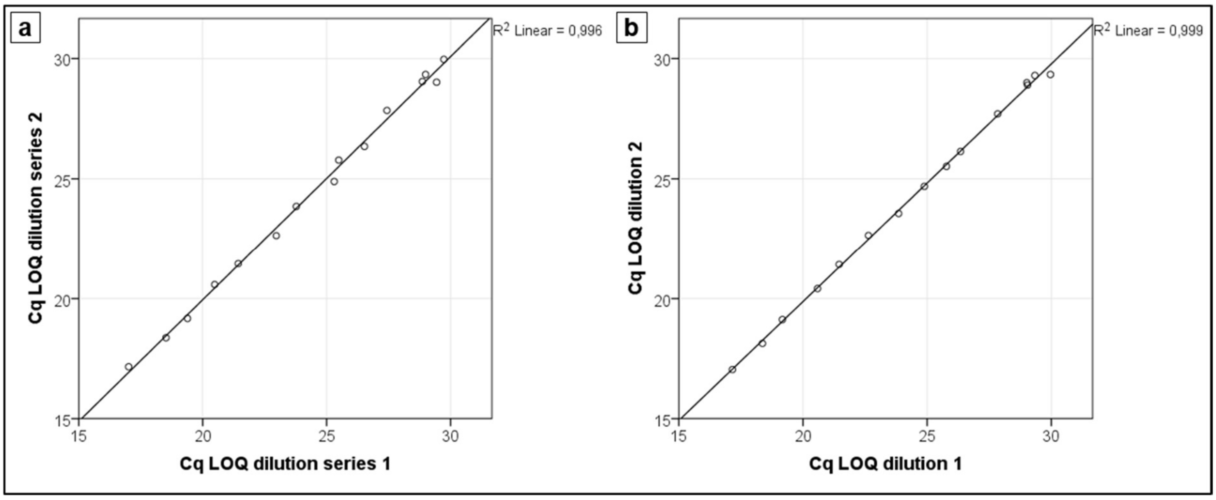

Supplementary Figure S13. Reproducibility of PNB-qPCR

a) Two PNB-qPCRs with two separate LOQ dilution series. The two LOQ measurements of two different dilution series diluted from the same stock of G12D mutated DNA fragments were in very high agreement (two-sided Pearson correlations, both ρ = 0.998 and $P < 10^{-16}$ for 15 comparisons each). b) Repeated dilution of the same first round products of a LOQ dilution series for PNB-qPCR. The results of a LOQ dilution series were identical after repeated dilution and second round qPCR of the first round product pools (two-sided Pearson correlation, ρ = 0.999, $P < 10^{-19}$ for 15 comparisons).

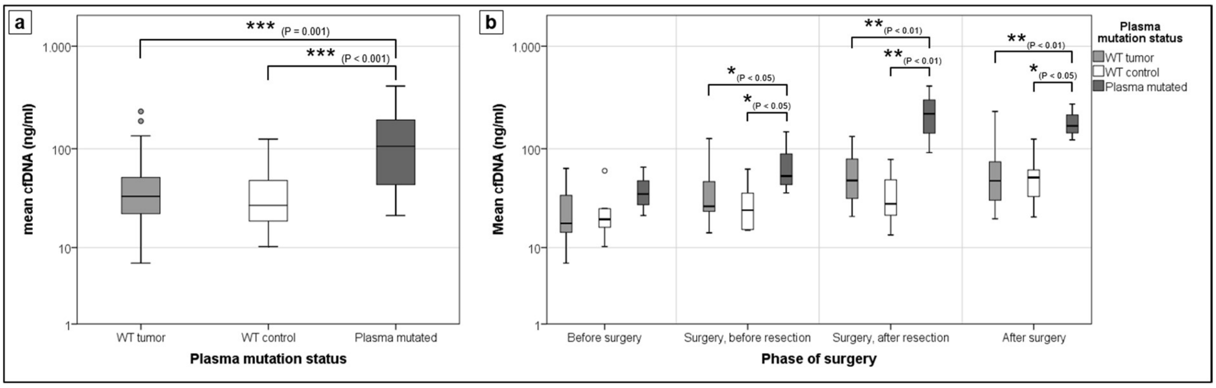

Supplementary Figure S14. Significance of plasma mutation status for cfDNA concentrations

cfDNA values of patients in whose plasma the *KRAS* point mutations had been detected were significantly higher than plasma of WT-patients for a) individually averaged concentrations over all four phases of the surgical process, and b) verified by a classification into the four main phases of the surgical process. Both Figures analyse the mean cfDNA concentrations of each patient in each phase.

a) Averaged cfDNA values of mutation positive patients (group M) were significantly higher than those of WT tumour patients (group T) and WT control patients (group C) (n = 112, df = 2, F = 18.97, P < 0.0001; mean cfDNA of M 88.2 ng/ml higher than T (95% CI, 49.3-127.1, P = 0.0001) and 101.0 ng/ml higher than C (95% CI, 60.5-141.5, P < 0.0001); both Tukey's HSD tests).

b) cfDNA concentrations of group M were significantly higher than those of groups T and C in periods II (P = 0.036, n = 28, mean difference M vs. T 38.7 ng/ml, 95% CI, -7.6-85.1 ng/ml, P = 0.04; mean difference M vs. C 50.3 ng/ml, 95% CI, 2.1-98.6 ng/ml, P = 0.042, test), III (n = 28, mean difference M vs. T 181.4, 95% CI, 94.3-268.5, P = 0.003; mean difference M vs. C 204.6, 95% CI, 113.9-295.2, P < 0.001), and IV (n = 28, mean difference M vs. T 116.5, 95% CI, 26.8-206.3, mean difference M vs. C 130.8, 95% CI, 37.4-224.3, P = 0.02 each; all Tukey's HSD tests).

Supplementary Tables

Supplementary Table S1. PCR primers

#	Primer	Direction	Sequence	Length	# rev. primer
1	Outer primer for	sense	GGCCTGCTGAAAATGACTGAATATAAACTTGTGG	34	2
2	Outer primer rev	antisense	TCATATTCGTCCACAAAATGATTCTGAATTAGCTG	35	
3	*KRAS* WT	sense	GAATATAAACTTGTGGTAGTTGGAGC	26	4
4	Inner reverse 1	antisense	TCTGAATTAGCTGTATCGTCAAGG	24	
5	Inner reverse 2	antisense	ATTAGCTGTATCGTCAAGGC	20	
6	G12D ARMS for	sense	AAACTTGTGGTAGTTGGAGCAGA	23	4
7	G12V ARMS for	sense	AAACTTGTGGTAGTTGGAGGTGT	23	4
8	G12A ARMS for	sense	ACTTGTGGTAGTTGGAGCAGC	21	4
9	G12S ARMS for	sense	ATAAACTTGTGGTAGTTGGAGATA	24	5
10	G12C ARMS for	sense	AAACTTGTGGTAGTTGGAGATT	22	5
11	G12R ARMS for	sense	AATATAAACTTGTGGTAGTTGGAGGTC	27	4
12	G13D ARMS for	sense	TGTGGTAGTTGGAGCTGGAGA	21	4
13	Sequencing primer	antisense	TCCAATCAAAATGCACAGAGA	21	
14	WT cloning for	sense	CTTAAGCGTCGATGGAGGAG	20	15
15	WT cloning rev	antisense	CAACAAAGCAAAGGTAAAGTTGG	23	
16	SDM rev	antisense	PHO-AAGTTTATATTCAGTCATTTTCAGCAGGC	29	
17	SDM G12D	sense	PHO-GTGGTAGTTGGAGCTGATGGCGTA	24	16
18	SDM G12A	sense	GTGGTAGTTGGAGCTGCTGGCGTA	24	16
19	SDM G12S	sense	GTGGTAGTTGGAGCTAGTGGCGTA	24	16
20	SDM G12C	sense	GTGGTAGTTGGAGCTTGTGGCGTA	24	16
21	SDM G12R	sense	GTGGTAGTTGGAGCTCGTGGCGTA	24	16
22	SDM G13D	sense	GTGGTAGTTGGAGCTGGTGACGTA	24	16
23	WT blocking primer	sense	ATAAACTTGTGGTAGTTGGAGCTGGTGGCG-NH3	30	-

Supplementary Table S2. qPCR probes

Probe	Direction	Sequence	Length	With Primer #
KRAS WT LNA probe	sense	[6FAM]CTC[+T][+T]GC[+C][+T]ACGC[+C]A[BHQ1]	15	3, 6-11
KRAS WT LNA Rev probe	antisense	[6FAM]GCGTA[+G]GC[+A][+A]G[+A]G[+T]G[BHQ1]	15	12

Supplementary Table S3. Running conditions of the ARMS primers

Mutation	Forward Primer	Reverse Primer	Annealing Temperature	Annealing Time
G12D	G12D ARMS for	Inner reverse 1	67 °C	30 seconds
G12V	G12V ARMS for	Inner reverse 1	67 °C	30 seconds
G12A	G12A ARMS for	Inner reverse 1	65 °C	40 seconds
G12S	G12S ARMS for	Inner reverse 2	65 °C	30 seconds
G12C	G12C ARMS for	Inner reverse 2	65 °C	40 seconds
G12R	G12R ARMS for	Inner reverse 1	65 °C	40 seconds
G13D	G13D ARMS for	Inner reverse 1	65 °C	30 seconds

Supplementary Methods

Blood sample treatment. Venous blood samples were collected one day prior to surgery, as well as directly before anaesthesia, under aesthetic, every 20 minutes during the course of surgery, and 3, 6, 24 and 72 hours after the end of surgery. After anaesthesia and during the course of surgery arterial blood samples were taken simultaneously. Blood samples of 1 ml per sample on the day of surgery and 6 ml per sample on the other days were taken in EDTA coated blood monovettes (Sarstedt, Nümbrecht, Germany). Blood plasma was separated in a first step within 45 minutes from blood withdrawal by centrifugation at 1,600 *x g* at 4 °C for 10 minutes. The obtained plasma was directly transferred to a new reaction tube and centrifuged again at 16,000 *x g* at 4 °C for 5 minutes. The supernatant was stored in a new reaction tube at -80 °C until further use.

cfDNA isolation from blood plasma. cfDNA was isolated from blood plasma using the QIA-amp Circulating Nucleic Acid Kit (Qiagen, Hilden, Germany) following the manufacturer's in-structions with the two following adjustments. Firstly, the carrier RNA provided in the kit was not added to the lysis buffer in the respective step, and secondly, water was used for elution instead of the provided elution buffer. Both adjustments were made due to better amplification results in the first round PCR in pre-tests and a better linearity of tested dilution series in the nested qPCR setting. cfDNA eluates were stored at -20 °C until further use.

cfDNA was isolated from all samples taken one day before surgery of all patients for mutation detection and samples from mutation positive patients at specific time points over the course of surgery.

cfDNA isolation from FFPE tissues. Formalin fixed paraffin embedded (FFPE) samples of the pathological tissues removed throughout the surgeries were tested for *KRAS* mutations. All FFPE tissue samples were approved by a pathologist. The FFPE tissues were deparaffin-ised with Roti®-Histol and DNA was isolated using the QIAamp DNA FFPE tissue Kit (Qiagen, Hilden, Germany) following the manufacturer's instructions. The obtained eluates were diluted

with water to concentrations of 1 ng/ µl due to observed reduction of the PCR efficiency in pre-tests when higher DNA concentrations had been inserted as templates.

Quantification of total cfDNA by qPCR. Total cfDNA was quantified by the three qPCR approaches described below. First, small volumes of all plasma samples were diluted and cfDNA was quantified by *L1PA2* qPCR directly from the diluted plasma sample. Thus, expenditure of time and money as well as a potential DNA loss of the isolation procedure were avoided. Total cfDNA in eluates of all samples that had been isolated for PNB-qPCR was quantified by two additional qPCR approaches. The first was a qPCR of the *LTR5* locus to determine the DNA loss during DNA isolation. The second was a mutation independent *KRAS* qPCR, which amplified all present *KRAS* fragments of exon 2 independent of the mutation status, to determine the percentage of mutated fragments. The comparison of the two latter qPCRs could have detected a possible copy number variation of the *KRAS* locus compared to total cfDNA[S1].

cfDNA was quantified by calculation based on the respective standard curves. qPCR results were calibrated based on positive controls of known DNA concentrations. All qPCRs were performed on a Bio-Rad CFX384 Touch™ Real-Time PCR Detection System (Bio-Rad, Munich, Germany).

***L1PA2*-qPCR.** cfDNA from all taken blood samples was quantified in unpurified plasma by qPCR of the *L1PA2* locus as described previously[18]. This qPCR employs a primer pair that targets a 90 bp long redundant sequence in the *L1PA2* element with approximately 3345 matches in the human genome, according to the USCS genome browser. It enables the detection and quantification of minimum fragments of the human genome without the risk of losing cfDNA through DNA purification. 6.4 µl of blood plasma that had been diluted beforehand 1:40 in H_2O were added to 41.6 µl of PCR mix consisting of a 2x concentrated TEGO buffer, Velocity polymerase (both Bioline, Luckenwalde, Germany), SYBR Green, FITC (both Sigma-Aldrich, Munich, Germany), $MgCl_2$ (Qiagen, Munich, Germany), as well as 0.34 µM of each the forward and the reverse primer. The qPCR was performed in triplicates of 5 µl each. The qPCR protocol consisted of an initial heating period of 2 minutes at 98° C, 35 cycles of 10 seconds at 94° C, 10 seconds at 64° C and 10 seconds at 75° C each, followed by a melt curve.

***LTR5* qPCR.** Isolated cfDNA was quantified by qPCR targeting the retroviral *LTR5* (long terminal repeat) locus, as described previously[19]. The primers target a 70 bp long sequence with approximately 195 matches in the human genome, according to the USCS genome browser. An in-house assay was used consisting of HotStarTaq *Plus* DNA Polymerase and the associated buffer and $MgCl_2$ (Qiagen, Munich, Germany), SYBR-Green, FITC (both Sigma-Aldrich, Munich, Germany), and dNTPs (Carl Roth, Karlsruhe, Germany). The detailed concentrations and protocol are described in Helmig *et al.*[19]. All tests were performed in triplicates.

***KRAS* WT qPCR.** *KRAS* WT qPCR targets exon 2 of the *KRAS* gene, employing a mutation unspecific primer pair targeting a 70 bp long sequence enclosing the mutation hotspots in codons 12 and 13. All tests were performed in triplicates. The final concentrations per well were 1x SsoAdvanced™ Universal Probes Supermix (Bio-Rad, Munich, Germany), 400 nM custom-built forward and reverse primer, 200 nM custom-built LNA probe, and 3.2 µl of template for a total of 8 µl per well. qPCR conditions were 2 minutes at 95 °C followed by 50 cycles of 5 seconds at 95 °C and 30 seconds at 67 °C and a final elongation step of 5 minutes at 72 °C.

Quantification of total DNA from FFPE-Samples. DNA isolated from FFPE samples was quantified by *KRAS* WT qPCR as described above for cfDNA quantification.

Quantification of ctDNA by PNB-qPCR - Primer design. For qPCR, we designed a *KRAS* wild type (WT) primer for whole cell free DNA (cfDNA) amplification and seven mutation specific primers using the amplification refractory mutation system (ARMS)[S2]. ARMS primers harbour the point mutation at the 3' end. Additionally, a mismatch base 3 to four base pairs from the 3' end further decreases the specificity of the primer for the WT-allele.

As it is known that circulating nucleic acids are highly fragmented, the amplified regions had to be as short as possible for high detection effectivity. For this purpose, we designed 15 bases short LNA probes for *KRAS* specific detection, each containing 5 locked nucleic acids (LNAs), enabling PCR fragment lengths between 59 and 70 base pairs. Primers for the first round PCR were located outside of the second round primers producing first round PCR products of 110 bp in total length. However, as the outer and the inner primers partly overlap for several base

pairs, the sequence between the 3’ ends of the two outer primers is only 41 bp long. Primer and probe constellations are displayed in supplementary Figure S1; primer and probe sequences are enlisted in supplementary Tables 2 and 3.

All primers were designed with the Primer3 program (http://bioinfo.ut.ee/primer3-0.4.0/; and http://bioinfo.ut.ee/primer3/ for the earlier version partly used for the described primers).

Standard preparation. To prepare constantly available homologue standards for all *KRAS* variants, plasmid vectors carrying a sequence surrounding the exon 2 of the human *KRAS* gene were inserted into competent E. coli bacteria and stored in liquid nitrogen. Site directed mutagenesis (SDM) was used to generate plasmids carrying the seven most common point mutations in *KRAS* exon 2, using primers that introduced the respective modified sequences. SDM was performed for all mutations except the G12V mutation, which was obtained from the homozygously mutated genomic DNA (gDNA) of the SW-480 cancer cell line (CLS, Eppelheim, Germany).

WT standard and G12V. First, we isolated human genomic DNA (gDNA) from whole blood with a salting out procedure[S3]. A PCR using the HotStar HiFidelity Polymerase (Qiagen, Hilden Germany) and a primer pair completely spanning *KRAS* exon 2 produced a DNA fragment of 895 bp. The PCR volume of 50 µl consisted of 10 µl of 5x Buffer containing dNTPs, 1 µl of 25mM $MgCl_2$, 1 µl of HotStar HiFidelity Polymerase, 31 µl of distilled H_2O, 6 µl of premixed primer mix, 5 mM each, and 1 µl template DNA, containing 400 ng of human genomic DNA. The PCR protocol was the following: 5 minutes at 95 °C, 40 cycles of 1 minute at 94 °C, 1 minute of 58 °C, and 1 minute of 72 °C, followed by 10 minutes of 72 °C. The PCR product was positively tested for the correct length by agarose gel electrophoresis. DNA was isolated with the NucleoSpin® Gel and PCR Clean-up Kit (Macherey-Nagel, Düren, Germany) and stored at -20 °C.

10 ng of the DNA products were ligated to linearized pCR®2.1 vector using the TA cloning Kit (Invitrogen, Carlsbad, CA) and subsequently transferred into freshly thawed competent E. coli bacteria following the manufacturer’s instructions. Ligation was performed overnight at 14 °C.

10-100 µl of the bacterial solution were plated on LB medium containing agar, X-gal, and Ampicillin and incubated overnight at 37 °C.

White colonies were incubated in LB medium containing Ampicillin. As a control for the insertion of the plasmid, we used a quick plasmid clean-up procedure, linearized the DNA by restriction enzyme digestion, and examined the resulting DNA by agarose gel electrophoresis. In case of a positive result, the bacterial stock was refilled to 5 ml with LB medium containing Ampicillin and re-incubated overnight at 37 °C. Plasmid DNA from 2-5 ml of the bacterial suspension were isolated with the QIAprep Spin Miniprep Kit (Qiagen, Hilden, Germany) following the manufacturer's protocol.

The resulting plasmid DNA was linearized by EcoR1 digestion following the manufacturer’s instructions, and sequenced at StarSeq (Mainz, Germany), using a primer spanning the region of interest in the *KRAS* gene. A fraction of the bacterial suspension and glycerine (30% final) containing the desired plasmid was frozen in liquid nitrogen.

The same procedure was used for isolation and transformation of gDNA from SW-480 tumour cells that are homozygous for the G12V point mutation.

Site directed mutagenesis. We introduced six of the seven most common point mutations in codons 12 and 13 of *KRAS* exon 2 into the plasmid sequences using site directed mutagenesis (SDM). The SDM primer carries the base of the point mutation in its centre to reduce the risk of misbinding. During SDM, we amplified the whole plasmid. For this purpose, the SDM primers had to start “back to back”, meaning that the 5' end of the forward primer was directly adjacent to the 5' end of the reverse primer.

For SDM PCR we used a Phusion® Hot Start Flex DNA Polymerase (NEB, Ipswich, MA) that creates blunt ends, which is essential for proper ligation in this setting, and features a low error rate of approximately 10^{-6} [S4]. 25 µl PCR mix contained the final concentrations of 0.01 U/µl Phusion® Hot Start Flex DNA Polymerase, 1x Phusion HF buffer, 0.5 mM $MgCl_2$ (all NEB, Ipswich, MA), 200 µM dNTPs, 500 nM premixed primer mix, 5 ng template DNA, and H_2O up to the volume of 25 µl. The PCR was performed on an Eppendorf PCR cycler for 30 seconds at

98 °C, 35 cycles of 10 seconds at 98 °C, 30 seconds at 70 °C, and 2.5 minutes of 72 °C, followed by 10 minutes of 72 °C.

All PCR products were digested with the DNA methylation specific restriction enzyme Dpn1 (NEB, Ipswich, MA), following the manufacturer's protocol, to eliminate the remaining WT template DNA fragments. The resulting DNA was Sanger sequenced with the same primer used for sequencing the *KRAS* wild type sequence (see above). Mutation positive DNA stocks were inserted into competent E. coli bacteria and stored as described above.

Primer establishment. All Primers were tested in heat gradients for annealing temperature and time, to optimize specificity and sensitivity. ARMS primers and the WT Standard primer were tested on a Bio-Rad CFX 384 real-time PCR cycler and all other primers were tested on an Eppendorf Mastercycler EP Gradient S PCR cycler.

Primers for mutation detection were optimized on three levels of PCR complexity, namely qPCR, nested qPCR, and pooled nested blocker qPCR (PNB-qPCR). qPCR contained ARMS primers and LNA probes. For nested qPCR a first round PCR was added, containing the outer primers and a newly designed and custom-built 3' amino *KRAS* WT blocking primer, as previously described by Lee et al.[8]. PNB-qPCR is a nested qPCR with pooled first round products (see below).

We determined the limits of detection and quantification (LOQ/ LOD) with standard dilution series of the respective WT or mutated *KRAS* fragments of known concentrations. All samples containing mutated DNA used for the standard curves and as positive controls were spiked with a background of 30,000 copies of WT *KRAS* DNA, corresponding to 100 ng of genomic DNA. The dilution series were created in 1:2 dilution steps from 12,000 to 0.8 copies per PCR equalling 28.5% to 0.003% mutated DNA. The initial stock was quantified with a NanoDrop 3300 fluorospectrometer using a PicoGreen assay (Thermo Scientific, Braunschweig, Germany). The dilution series for *KRAS* WT was performed with gDNA previously quantified with a NanoDrop 1000 starting with 100 ng in 1:2 dilution steps.

qPCR. Temperature and length of the annealing steps of all ARMS primers were optimized separately for combinations of highest sensitivity and qPCR efficiency. All qPCRs were performed with a two-step protocol of 2 minutes at 95 °C, followed by 50 cycles of 5 seconds at 98 °C and 30-40 seconds annealing, and a final elongation step of 5 minutes at 72 °C. All qPCRs were performed in triplicates, except for the final LOQ/LOD-experiments that were performed in seven replicates. Final concentrations were 1x SsoAdvanced™ Universal Probes Supermix (Bio-Rad, Munich, Germany), 400 nM forward ARMS and reverse primer, 200 nM LNA probe, and 3.2 µl of template for a total of 8 µl per well. The final running conditions for the used primer pairs are listed in Supplementary Table 3. All qPCRs were performed on a Bio-Rad CFX384 Touch™ Real-Time PCR Detection System and analysed with the corresponding software (Bio-Rad, Munich, Germany).

Nested qPCR. To enrich mutated fragments, reduce background noise, and enhance the concentration of DNA in the template, we expanded the qPCR by a first round PCR making use of a 3’ C6 amine modified WT blocking primer. Final concentrations of the first round PCR were 0.002 U/µl Phusion® Hot Start Flex DNA Polymerase, 1x Phusion HF buffer, 0.5 mM $MgCl_2$ (all NEB, Ipswich, MA), 200 µM dNTPs, 1 µM blocking primer, 400 µM outer primer mix, and 14 µl of template and H_2O for a total of 25 µl. The first round PCR can be adapted to varying volumes up to 200 µl, permitting up to 112 µl of template. The PCR protocol was performed as follows: 30 seconds at 98 °C, 20 cycles of 10 seconds at 98 °C and 30 seconds at 69 °C, followed by 5 minutes of 72 °C. The PCR product was diluted 1:50 in H_2O (2 µl product plus 98 µl H_2O) and served as template for the second round qPCR.

The first round PCR was established for the exact annealing temperature, at which the WT blocking primer has a significantly higher affinity to the WT sequence, but a lower affinity to possible mutated sequences, than the regular forward primer.

PNB-qPCR. The first round PCR and the subsequent dilution step considerably increased the variation of the outcome. Thus, the quantification efficiency might be reduced even though the template for the qPCR is enriched. Therefore, we added an additional pooling step to reduce

this variation. First round PCRs were run in quintuplets. 8 µl of each PCR product were transferred and pooled in a new reaction tube. This pool was subsequently diluted 1:50 for use in the second round qPCR. Figure 2 illustrates the workflow of the PNB-qPCR.

DNase 1 activity assay. To investigate whether the changes in cfDNA concentration might be reflected in DNase 1 activity, as reported before for physical stress induced by exercise[S5], a DNase 1 activity reduction ELISA assay was performed following the manufacturer's instructions (ORGENTEC, Mainz, Germany). The assay was performed with plasma of all samples of ctDNA positive patients that had been analysed by PNB-qPCR.

Statistics. Statistical evaluations were performed with SPSS (version 22) or JMP (version 11) statistical software. All correlations were two-sided Pearson’s correlations unless specified otherwise.

Supplementary Results

qPCR establishment. The qPCRs using the ARMS primers were established to LOQs between 50 and 6.25 copies per qPCR (LOQs: G12D 12.5 copies, G12V 50 copies, G12A 12.5 copies, G12S 50 copies, G12C 6.25 copies, G12R 25 copies, G13D 6.25 copies; mean 23.2 copies, median 12.5 copies; Supplementary Fig. S2, Supplementary Fig. S4). The limit of detection (LOD) was in most cases determined by the false positive signals generated by the added high background of WT fragments, which were higher than the Y-intercept of the standard curves (calculated copies of false positive signals for 30,000 WT copies: G12D 7.3 copies, G12V 14.5 copies, G12A 0.14 copies, G12S 24 copies, G12C 1.13 copies, G12R 0.36 copies, G13D 1.76 copies; mean 7.03 copies, median 1.76 copies, Supplementary Fig. S4).

Nested qPCR establishment. The first round PCR utilizing a WT-specific 3'-C6 amine blocking primer improved the qPCR as a detection method for mutated DNA in three ways. Firstly, the enrichment of the mutated fragments in the qPCR template allows for higher sensitivity due to a higher number of targets. Secondly, the simultaneous reduction of background noise generated by WT fragments in the same template assures a higher specificity. Thirdly, the amount of template for the performed qPCRs is greatly enlarged, permitting over 100 times more qPCR detections.

Therefore, instead of using the blocking primer directly in the qPCR, we inserted it into a first round PCR containing mutation unspecific *KRAS* outer primers. The differences induced by addition of the blocking primer ranged from 1.1 to 4 cycles for the mutations (G12D 1.1 cycles, G12V 2.4 cycles, G12A 3.98 cycles, G12S 1.75 cycles, G12C 1.94 cycles, G12R 1.87 cycles, G13D 3.29 cycles; mean 2.33 cycles, median 1.94 cycles) and 12.79 cycles for WT, adding up to an enrichment between 8.8 and 11.7 cycles (G12D 11.69 cycles, G12V 10.39 cycles, G12A 8.8 cycles, G12S 11.04 cycles, G12C 10.85 cycles, G12R 10.92 cycles, G13D 9.5 cycles; mean 10.45 cycles, median 10.85 cycles). These cycle differences correspond to an enrichment of mutated fragments between 446-fold to 3304-fold in a PCR with an efficiency of 1.

The absolute enrichment of mutated fragments by the 20 cycles first round PCR (2^Cq$_{qPCR}$–Cq$_{nested\ qPCR}$) was on average 109-fold, ranging from 21-fold to 588-fold (G12D 8.09 cycles, G12V 7.44 cycles, G12A 5.15 cycles, G12S 9.17 cycles, G12C 6.07 cycles, G12R 7.61 cycles, G13D 4.35 cycles; mean 6.77 cycles, median 7.44 cycles) (Supplementary Fig. S3), favouring low-copy detection.

The probability of false positives [2^Cq(false pos.)$_{nested\ qPCR}$–Cq(false pos.)$_{qPCR}$] was reduced in all cases (4.7-fold to 243.3-fold, mean 73.7-fold, median 28.3-fold) to below 0.5 copies in all cases (calculated copies of false positive signals for 30,000 WT copies: G12D 0.03 copies, G12V 0.09 copies, G12A 0.03 copies, G12S 0.45 copies, G12C 0.04 copies, G12R 0.03 copies, G13D 0.13 copies; mean 0.11, median 0.04). Therefore, the difference between possible false positive signals and the signal for a single copy is greater than one qPCR cycle for all mutations.

However, the LOQs of the standard curves could not be improved. They were unaltered for four primer pairs (G12D, G12V, G12S, G12R) and impaired for the other three primer pairs (G12A 25 copies, G12C 25 copies, G13D 25 copies; mean all 30.2 copies, median 25 copies; Supplementary Fig. S4), compared to the single round qPCR.

Thus, the nested qPCR was superior in detection of minute amounts of mutated DNA fragments but by trend inferior in quantifying them, compared with qPCR.

PNB-qPCR establishment. Pooling products from five first round PCRs with the same input template reduced variation induced by the first round PCR and improved quantification accuracy. At the same time, a higher variation of total input volume was facilitated. To investigate the best number of first round PCRs per step, we pooled three, five and seven PCR products in a dilution series. The results showed that the optimal improvement is reached at pools of five products, whereas pools of seven did not further improve quantification sensitivity (Supplementary Fig. S5).

Using PNB-qPCR we could considerably lower the LOQs for all primer pairs compared with qPCR and nested qPCR except for the G13D primer pair (LOQs: G12D 3.1 copies, G12V 12.5

copies, G12A 3.1 copies, G12S 6.25 copies, G12C 3.1 copies, G12R 12.5 copies, G13D 12.5 copies; mean 7.6 copies, median 6.25 copies; Supplementary Fig. S3, Supplementary Fig. S4). The limit of detection remained the same as in the nested qPCR, allowing for a clear discrimination between mutation positive and mutation negative samples by single copy detection.

Repetition of the whole LOQ-determination process and also of the second part, from the dilution step on, showed high reproducibility of the method (Supplementary Fig. S13)

Error rate of the polymerases. To reduce the error rate of our nested PCR assay we chose two polymerases with a fidelity as high as possible. In the first round PCR a high fidelity Phusion® Hot Start Flex DNA Polymerase was used with a reported error rate of $4.2*10^{-7}$ [S6] to $2.6*10^{-6}$ [S7]. The error rate of the second round PCR is slightly higher with a polymerase error rate of $2.79*10^{-5}$ [S8] to $2.04*10^{-6}$ [S4]. This higher error rate is counterbalanced by the use of triplets in the qPCR and the high amount of template for the second round generated by the first round PCR and the following dilution step allowing for repetitions of the PCR in case of questionable results.

Supplementary references

S1. Beroukhim, R. *et al.*, *The landscape of somatic copy-number alteration across human cancers*, Nature 463(7283):899-905 (2010)

S2. Newton, C. R., *Analysis of any point mutation in DNA. The amplification refractory mutation system (ARMS)*, Nucleic Acids Res. 17(7):2503-16 (1989)

S3. Miller, S. A., Dykes, D. D., Poleski, H. F., *A simple salting out procedure for extracting DNA from human nucleated cells*, Nucleic Acids Res. 16(3):1215 (1988)

S4. Lee, J. I., Cho S. S., Kil, E.-J., Kwon, S-T., *Characterization and PCR application of a thermostable DNA polymerase from Thermococcus pacificus*, Enzyme and Microb Technol. 47(4) 147-152 (2010)

S5. Beiter, T. *et al.*, *Neutrophils release extracellular DNA traps in response to exercise*, J of Appl Physiol. 117(3):325-33 (2014)

S6. Li, M., Diehl, F., Dressman, D., Vogelstein, B., Kinzler, K. W., *BEAMing up for detection and quantification of rare sequence variants*, Nat Meth. 3(2):95-7 (2006)

S7. McInerney, P., Adams, P., Hadi, M. Z., *Error Rate Comparison during Polymerase Chain Reaction by DNA Polymerase*, Mol Biol Int. 2014:287430 (2014)

S8. Ppyun, H., *Improved PCR performance using mutant Tpa-S DNA polymerases from the hyperthermophilic archaeon Thermococcus pacificus*, J Biotechnol. 164(2):363-70 (2012)
